# Comment on “Care Coordination Experiences of Whole Families Caring for Children With Medical Complexity in Rural Areas”

**DOI:** 10.1111/hex.70916

**Published:** 2026-10-08

**Authors:** Anneliese de Groot, Karen Hutchinson, Amy Hickman, Raghu Lingam, Yvonne Zurynski

**Affiliations:** ^1^ Australian Institute of Health Innovation Macquarie University Macquarie Park New South Wales Australia; ^2^ School of Population Health University of NSW Kensington New South Wales Australia; ^3^ Central Coast Local Health District NSW Health Gosford New South Wales Australia; ^4^ Flinders University Bedford Park South Australia Australia; ^5^ School of Clinical Medicine University of NSW Randwick New South Wales Australia

Dear Editor,

We thank the authors of a letter commenting on our recent article [1], in which they discussed opportunities to evaluate care coordination as a family‐level intervention. We agree with the perspectives presented, exploring how family‐focused evaluation methods could help to establish intervention‐related changes, providing actionable evidence to better inform whole family support in the future. However, we propose ethical and logistical considerations to support planning for family‐focused research and evaluation, including reducing burden on family members' time and capacity, avoiding overwhelm and distress, and allowing families to shape the research direction and identify what is important to them.

As described in the letter responding to our article, family‐level evaluation methods could include conducting repeated interviews with multiple family members, at baseline and post‐exposure. However, each research interaction poses an additional burden for families who are time‐poor, overloaded with healthcare coordination and administration tasks, and worried about their children [2]. Data sources that are independent of families' resources, that is, observation, could be utilised wherever possible to avoid increasing participants' logistical load [3]. Flexible and generous research timeframes can also accommodate family members' changing schedules and needs during recruitment and data collection [3, 4, 5]. Additionally, consent (or assent in the case of children) should be explicitly treated as an ongoing voluntary process to ensure all family participants are empowered through their participation [3, 5].

We agree that pragmatic recruitment and data collection methods in our study likely reduced the representation of the experiences of families under greatest strain [1]. It is important to understand the experiences of families in crisis, which may assist in the identification of unique experiences, support needs and strategies to avert crisis in the future [4]. For families experiencing distress, familiarity and rapport with research staff, i.e. embedding researchers within clinical services, may improve trust and the likelihood of families participating in research [4]. However, establishing relationships between researchers and participants increases the stakes for maintaining clarity and transparency relating to research roles, processes and expected outcomes to ensure ethical integrity [3, 4]. Families experiencing significant levels of stress, distress or crisis have limited capacity to participate in research, regardless of the best intentions of researchers. Family autonomy to choose participation or not must be respected at all times. Recruitment through clinical settings must also avoid jeopardising the critically important trusting relationships between families and clinicians, which must always take precedence over research.

To balance any risk of research burden on families caring for children with medical complexity, it is important to increase the potential for the research to create real value in families' lives. Many families choose to participate to create benefits for other families in similar circumstances in the future, rather than for themselves [2]. To increase this benefit and value for whole families, researchers should partner with multiple family members to co‐design evaluations, selecting outcomes that address families' priorities amongst those identified by other interest‐holders [5].

## Author Contributions

**Anneliese de Groot:** conceptualisation, writing – original draft, writing – review and editing. **Karen Hutchinson:** writing – review and editing. **Amy Hickman:** writing – review and editing. **Raghu Lingam:** writing – review and editing. **Yvonne Zurynski:** writing – review and editing.

## Funding

The authors have nothing to report.

## Conflicts of Interest

The authors declare no conflicts of interest.

## Data Availability

Data sharing is not applicable to this article as no datasets were generated or analysed during the current study.

## References

[1] A. de Groot , K. Hutchinson , A. Hickman , et al., “Care Coordination Experiences of Whole Families Caring for Children With Medical Complexity in Rural Areas,” Health Expectations 29, no. 4 (August 2026): e70793, 10.1111/hex.70793. PMC13431284 42545824

[2] J. M. Nathe , T. T. Oskoui , and E. M. Weiss , “Parental Views of Facilitators and Barriers to Research Participation: Systematic Review,” Pediatrics 151, no. 1 (2022): e2022058067, 10.1542/peds.2022-058067. PMC9808610 36477217

[3] M. Miller‐Day and V. S. Katz , “Unique Ethical Challenges in Qualitative Interviews With Families and Children,” Methods in Psychology 14 (January 2026): 100250, 10.1016/j.metip.2026.100250.

[4] T. L. Morgan , O. E. Clark , S. Whiteside , et al., “Recruiting Families and Children Newly Diagnosed With Cancer for Behavioral Research: Important Considerations and Successful Strategies,” Psycho‐Oncology 28, no. 4 (April 2019): 928–930, 10.1002/pon.5012. 30702782

[5] A. E. Butler , J. Appleyard , K. Steadman , et al., “Working Together to Design Research for the Paediatric Intensive Care Unit: A Practical Example of Researcher–Family Partnerships for Paediatric Intensive Care Unit Research,” Australian Critical Care 39, no. 4 (January2026): 101629, 10.1016/j.aucc.2026.101629. 42492339

